# Bioactive Metabolites of *Solanum tuberosum* L. in Inflammation Modulation and Tissue Regeneration: A Scoping Review of Wound Healing Mechanisms

**DOI:** 10.3390/biomedicines14081758

**Published:** 2026-08-04

**Authors:** Wahyu Hidayat, Mieke Hemiawati Satari, Ronny Lesmana, Solachuddin Ichwan, Irna Sufiawati

**Affiliations:** 1 Doctoral Study Program in Medical Science, Faculty of Medicine, Universitas Padjadjaran, Sumedang 45363, Indonesia; wahyu.hidayat@unpad.ac.id; 2 Department of Oral Biology, Faculty of Medicine, Bandung Islamic University, Bandung 40116, Indonesia; satarimieke15@gmail.com; 3 Department of Biomedical Sciences, Faculty of Medicine, Universitas Padjadjaran, Sumedang 45363, Indonesia; ronny@unpad.ac.id; 4 Dentistry Program, PAPRSB Institute of Health Sciences, Universiti Brunei Darussalam, Gadong BE1410, Brunei; solachuddin.ichwan@ubd.edu.bn; 5 Department of Oral Medicine, Faculty of Dentistry, Universitas Padjadjaran, Sumedang 45363, Indonesia

**Keywords:** bioactive metabolites, flavonoids, inflammation, *Solanum tuberosum* L., scoping review, tissue regeneration, wound healing

## Abstract

Background/Objectives: Persistent inflammation and impaired tissue regeneration delay mucosal and cutaneous wound healing. Potato (*Solanum tuberosum* L.) preparations contain bioactive metabolites with anti-inflammatory and regenerative potential, yet their wound-healing mechanisms have not been systematically mapped. This scoping review aimed to map potato-derived bioactive metabolites and their roles in modulating inflammation and promoting tissue regeneration during wound healing. Methods: A scoping review was conducted in accordance with the PRISMA-ScR guidelines. Articles published between 2015 and 2025 were retrieved from the Scopus, PubMed, EBSCO, and ScienceDirect databases combined with hand searching. Eligible studies included in vivo animal models; mechanistically relevant in vitro studies; and limited clinical or human skin safety studies investigating potato-derived extracts, fractions, or defined preparation effects in wound, ulcer, and burn healing. Results: Among the 583 records identified, only 15 met the inclusion criteria. These studies reported five major bioactive metabolite classes—flavonoids/anthocyanins, polyphenols, alkaloids, polysaccharides, and peptides—together with potato-derived exosomal vesicles as an extracellular vesicle/delivery system (not a metabolite class). Potato-derived preparations have been associated with accelerated wound closure, enhanced fibroblast proliferation, collagen deposition, and the attenuation of inflammatory mediators. Molecular evidence, which was included in only six studies, supported the modulation of NF-κB, STAT1/3, COX-2, iNOS, cytokines, and MMP-9. In addition, five priority gaps were identified: lack of standardization, fragmented biomarker profiling, limited compound-specific validation, scarce clinical translation, and absence of omics-based and complex wound models. Conclusions: Potato-derived bioactive metabolites show multi-target preclinical associations with wound healing and warrant further development as potential adjunctive therapies for mucosal and cutaneous wounds after standardization and clinical validation.

## 1. Introduction

Medicinal plants have been used to treat a range of diseases since ancient times. Their healing properties are attributed to naturally synthesized secondary chemical metabolites. Notably, the richest sources of active metabolites are seeds, bark, roots, leaves, and fruits [1]. In addition, herbal medicines play a crucial role in accelerating wound healing and creating a conducive environment for ulcer healing. Ulcers are crater-like lesions of the mucosa or skin with epithelial loss and may be caused by mechanical, iatrogenic, chemical, or thermal factors; open wounds are susceptible to infection and delayed closure when inflammation, oxidative stress, and impaired extracellular matrix (ECM) remodeling persist [1,2].

After an injury occurs, wound healing proceeds through four overlapping phases: (1) hemostasis, which occurs immediately after injury; (2) inflammation, characterized by swelling and mediator release; (3) proliferation, during which new tissue and blood vessels form; and (4) remodeling, when tissue architecture is restored [1,2,3,4]. Recurrent injuries, infections, insufficient oxygenation, and free radical formation can hinder healing and promote chronic wound development. Conventional topical therapies include povidone-iodine and corticosteroids. However, long-term corticosteroid use may cause local and systemic adverse effects. Recent advances in wound-repair biomaterials further illustrate this unmet need; for example, dendritic hydrogels with inherent antibacterial activity have been developed for bacteria-infected wounds [5], and modular bioadhesives that immobilize epidermal growth factor have accelerated full-thickness wound healing in preclinical models [6]. These innovations highlight progress in infected and regenerative wound care, yet potato-derived metabolite evidence remains separately mapped and chemically under-standardized. Consequently, natural products with antibacterial, antioxidant, and anti-inflammatory properties have been increasingly investigated as adjunctive wound care strategies [1,4].

The development of herbal therapeutics for skin ulcers and oral ulcers is particularly important, especially in resource-limited settings. Many bioactive metabolites from tropical plants exhibit anti-inflammatory activity and support tissue regeneration. *Solanum tuberosum* L. (potato; Solanaceae) is a herbaceous plant that produces edible tubers from enlarged underground stems, not from roots [7,8]. The cultivated potato grown worldwide is mainly *S. tuberosum* ssp. *tuberosum*, whereas related forms in the Andes are better suited to seasons with short daylight [8]. This plant usually grows in a low to somewhat upright form, has leaves composed of several leaflets, flowers that are symmetrical from all directions and appear star-shaped, and tubers with small buds called “eyes”; potatoes are generally propagated by planting the tubers. The potato originated near Lake Titicaca in the Andes approximately 8000 years ago and then spread to many countries as a daily staple food and an industrial raw material [4,8]. For wound-healing research, tuber flesh and peel are most important because they contain starch, minerals, vitamins, and antioxidant substances from the phenolic group. In contrast, green aerial parts may contain higher levels of toxic glycoalkaloids [7,8].

Potato tubers and peels are rich in antioxidant substances from the phenolic acid group—especially chlorogenic acid—together with flavonoids and purple pigments (anthocyanins) that are more abundant in colored potatoes. Potato also contains characteristic Solanum compounds, namely α-solanine and α-chaconine, which warrant attention because they are pharmacologically active and can be toxic when present in excess [4,7,8,9,10]. About half of potato phenolics are located in the peel and nearby tissues. Therefore, the peel may be used as a source of antioxidant compounds [9,10,11]. In traditional medicine, raw potato juice, potato slices, and potato dressings have been used for digestive disorders, fever, pain, gastric inflammation, skin inflammation, burns, and wound care [4,8]. Modern studies link potato preparations to antioxidant, antimicrobial, anti-ulcer, and anti-inflammatory effects [4,7,8,12]. Potato has also been described as a food that may suppress inflammation because it contains starch that is difficult to digest, fiber, and purple pigments (anthocyanins); however, most of this evidence comes from intestinal or whole-body studies rather than from studies of skin or oral wounds, so the findings should be interpreted cautiously in this review [13].

Within this botanical and chemical context, potato peel dressings have been reported in clinical wound care, including severe soft-tissue infection (necrotizing fasciitis) [14] and minor burns [15]. This increases interest in developing potato as a therapy, not only in animal research. Tiarasanti et al. [16] and Santoso et al. [17] reported that potato peel ethanol extract gel accelerates gingival and palatal wound healing in rats. These findings indicate that bioactive metabolites and related preparations derived from potato can influence both the inflammatory phase and the regeneration phase of tissue repair.

Previous reviews have emphasized the in vitro pharmacological activities of potato metabolites, their antioxidant contents, and their nutritional significance [4,7,8,9,10,11,12,13,18,19,20,21,22]. However, to the best of our knowledge, no scoping or systematic review has comprehensively mapped in vivo and mechanistically relevant in vitro evidence on potato-derived extracts in wound healing, with explicit focus on inflammation modulation, tissue regeneration, and molecular pathways, such as NF-κB, COX-2, and matrix metalloproteinases (MMPs) pathways. Although promising findings exist, the published research remains heterogeneous in terms of potato varieties, plant parts, extraction methods, formulations, animal models, and outcome measures. Methodological variability limits comparability and slows translational development.

The following five critical knowledge gaps justify the present review. First, there are no operational standards for the variety, plant parts, solvents, formulations, or doses of potato-based wound therapies. Second, the molecular evidence is fragmented; most wound healing studies rely on macroscopic or histopathological endpoints, whereas only a minority report inflammatory and regenerative biomarkers. Third, causal attribution to specific bioactive compounds (e.g., *p*-coumaroyl anthocyanins [CAJY], chlorogenic acid, glycoalkaloids, and potato polysaccharides [STPs]) remains limited because most studies have used crude extracts. Fourth, clinical evidence is scarce, and no randomized controlled trials (RCTs) have evaluated standardized potato extract formulations against conventional wound care agents in humans. Fifth, multidisciplinary approaches, including network pharmacology, metabolomics, and complex wound models (diabetic, infected, or chronic wounds), have not been applied to potato extract research.

Therefore, this scoping review aimed to map the bioactive metabolites of *Solanum tuberosum* L. and their roles in modulating inflammation and promoting tissue regeneration during wound healing. Specifically, to summarize the existing literature, we addressed the following research questions using the PCC framework: “What bioactive metabolites have been identified in potato peel and tuber extracts used in wound-healing research? What experimental models and outcomes have been used to evaluate the anti-inflammatory and regenerative effects of potato-derived extracts? What molecular pathways and biomarkers mediate the wound healing effects of potato metabolites? What methodological, mechanistic, and translational gaps remain for future research and clinical development?” By synthesizing evidence from the included studies, this review provides an evidence map, a multi-target mechanistic framework, and a structured future research agenda to support the development of standardized, evidence-based, potato-derived wound therapies.

## 2. Methods

### 2.1. Design

This scoping review followed the methodological framework of Arksey and O’Malley, as refined by Levac et al. [23,24], and was reported in accordance with the Preferred Reporting Items for Systematic Reviews and Meta-Analyses extension for Scoping Reviews (PRISMA-ScR) [25]. Study selection was illustrated using the PRISMA 2020 flow diagram for new systematic reviews [26]. The protocol was not prospectively registered with PROSPERO because it was a scoping review; however, the eligibility criteria, search strategy, and recording procedures were predetermined before screening and fully documented. The scoping review design was chosen because the included studies were heterogeneous in terms of study design (in vivo or in vitro models and limited clinical evidence), intervention format (crude extracts, isolated fractions, exosomal vesicles/delivery systems, and dressings), and outcome measures (macroscopic, histopathological, and molecular). This approach allows for a broad mapping of the available evidence, the identification of research gaps, and the formulation of future research agendas. The review process included five stages: (1) formulating the research question, (2) identifying relevant studies, (3) selecting studies based on predefined criteria, (4) data extraction, and (5) synthesis and reporting of findings.

### 2.2. Eligibility Criteria (PCC Framework)

Eligibility was defined using the PCC framework: the population included bioactive metabolites from *Solanum tuberosum* L. (peel, tuber, or defined fractions thereof); the concept was their role in the modulation of inflammation and/or promotion of tissue regeneration in wound healing; and the context was experimental or clinical studies evaluating potato-derived preparations in wounds, ulcers, burns, or relevant inflammatory repair models. The search was limited to papers in English and Indonesian due to the linguistic skills of the study team.

The inclusion criteria were as follows: Original peer-reviewed articles published between January 2015 and December 2025; full text available in English or Indonesian; studies either investigating potato-derived extracts, fractions, or defined preparations for wound healing, mucosal ulcer repair, and burn healing or using directly relevant inflammatory regeneration models (including in vivo and mechanistically relevant in vitro models and clinical studies). Exclusion criteria: Narrative or systematic reviews; conference abstracts without full methods; duplicate publications; studies unrelated to wound healing, inflammation, or tissue regeneration; and studies without a sufficient methodological description.

### 2.3. Information Sources and Search Strategy

Literature searches were conducted using the Scopus, PubMed, EBSCO, and ScienceDirect databases from January 2015 to December 2025, combined with targeted hand searching. The search strategy combined the following terms, related to potatoes, wound healing, and bioactive metabolites: (“*Solanum tuberosum* L.” OR “potato”) AND (“wound healing” OR “ulcer” OR “burn wound”) AND (“extract” OR “bioactive metabolites” OR “phytochemical”).

Synonyms and related terms (e.g., inflammation, tissue regeneration, peel extract, and anthocyanin) were added iteratively. The reference lists of the included articles and prior reviews were manually searched to identify additional eligible studies. As previously mentioned, the language was limited to English and Indonesian, based on the proficiency of the reviewing team. The full per-database search strategies are provided in the Supplementary Materials.

### 2.4. Study Selection

Two independent reviewers screened the titles and abstracts, and then assessed the full texts against the eligibility criteria. Disagreements were resolved through discussion and are presented in the results section. A PRISMA-ScR flow diagram based on the PRISMA 2020 template summarizes the identification, screening, eligibility assessment, and inclusion steps.

**Figure 1:**
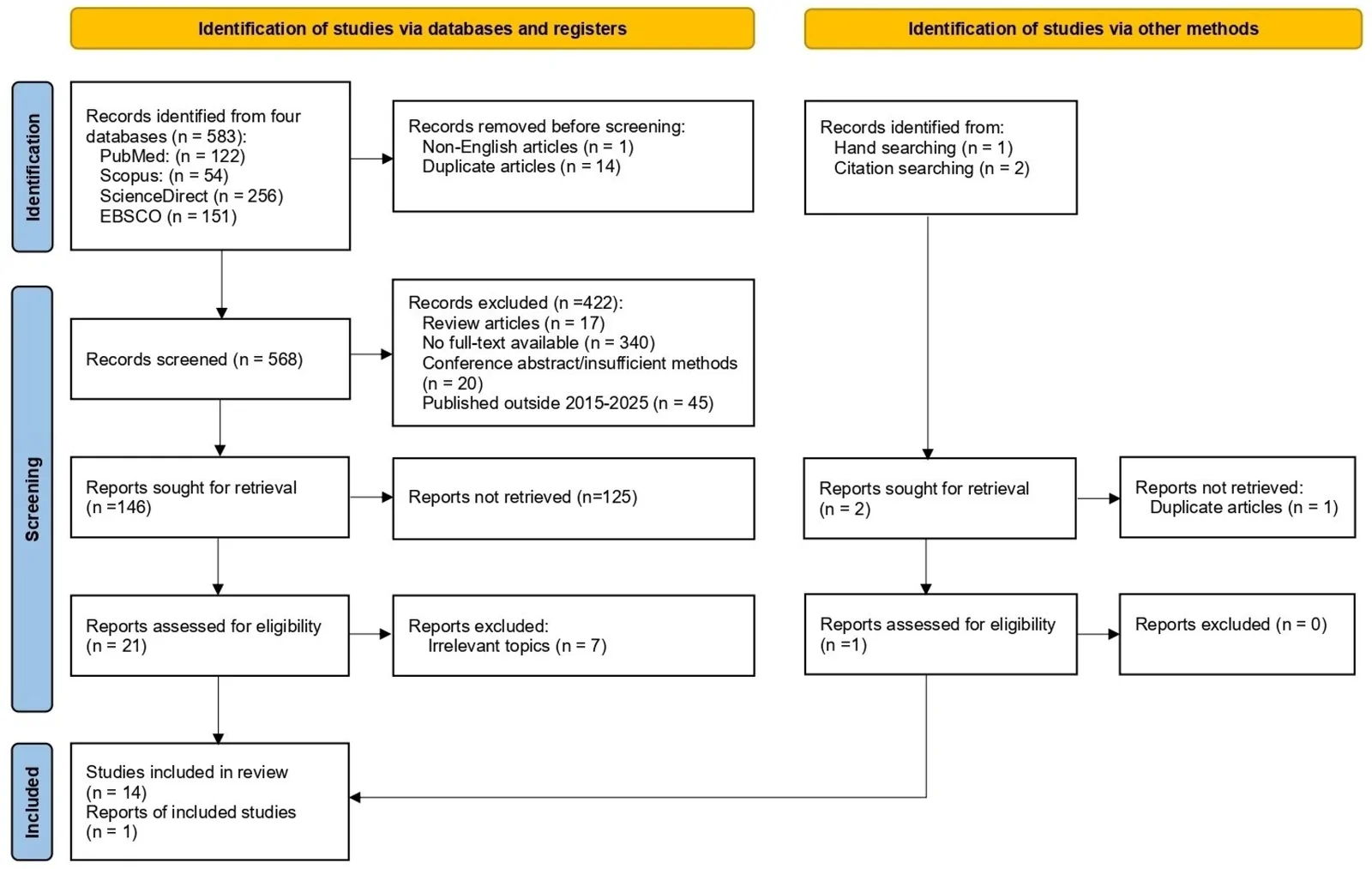
PRISMA-ScR flow diagram (PRISMA 2020 template) depicting the study identification, screening, eligibility assessment, and inclusion steps.

A total of 583 records were identified from four databases: PubMed (*n* = 122), Scopus (*n* = 54), ScienceDirect (*n* = 256), and EBSCO (*n* = 151). Before screening, 15 records were removed (one non-English article and 14 duplicates), leaving 568 records for title and abstract screening. Of these, 422 records were excluded (review articles, *n* = 17; not full text or not related to wound healing, *n* = 340; conference reports or insufficient methods, *n* = 20; outside the 2015–2025 window, *n* = 45). Thus, 146 reports were sought for retrieval; 125 were not retrieved (not eligible research articles or studies not using potato extracts, *n* = 47; in vivo/in vitro studies not related to wound-healing mechanisms, *n* = 78). Twenty-one full-text reports were assessed for eligibility; seven were excluded after full-text review (irrelevant topics, *n* = 7). Fourteen studies met the inclusion criteria from database searches. Additionally, one record was identified through hand searching and two through citation searching. After removing one duplicate, one report was assessed for eligibility, resulting in one additional included study from hand searching. In total, 15 studies were included (Figure 1).

### 2.5. Data Charting and Extraction

A standardized data-charting form was developed and piloted on three studies. The extracted variables included first author and year, potato variety, plant part, extraction solvent and method, formulation and dose, study design (in vivo, in vitro, or clinical), experimental model, phytochemical classes reported, anti-inflammatory and regenerative outcomes, molecular biomarkers, level of compound identification (crude extract, fraction, or isolated compound), and key findings.

Data charting was performed by two reviewers and verified for consistency. As this is a scoping review, a formal risk-of-bias scoring was not conducted; however, the study design and reporting limitations were considered during data synthesis.

### 2.6. Data Synthesis and Analysis

This study used a descriptive and thematic synthesis approach. Quantitative mapping included: (1) distribution by publication year, potato plant parts used, and study design; (2) categorization of bioactive metabolite classes, preparations, and delivery systems; (3) outcome grouping by wound type and healing phase; and (4) evidence mapping of reported molecular targets (NF-κB, COX-2, iNOS, STAT, MMPs, cytokines) against metabolite classes.

To determine the evidence confidence for the mechanistic claims, data were analyzed qualitatively and structured using thematic synthesis in accordance with PRISMA-ScR thematic standards [23,24,25], and the principles of evidence triangulation and indirectness from the GRADE approach were adapted [27]. Because heterogeneous preclinical designs preclude formal GRADE assessments of clinical outcomes, the assigned grades reflect only the certainty of pathway-level mechanistic claims. A strong confidence/certainty grade was assigned when ≥3 independent included studies reported the same molecular target with explicit measurement (protein, mRNA, or promoter activity) or when a chemically defined isolate demonstrated direct pathway validation. A moderate grade was assigned when two independent studies (either using the same kind of models or one in vivo and one in vitro model) addressed the same target without corroboration from a third study. A weak grade was assigned when the evidence was derived from a single study, from in vitro inflammation models without direct wound endpoints, or from crude extract testing without molecular confirmation. Grades were downgraded when preparations, models, or metabolite classes were heterogeneous, thereby limiting the causal attribution. Importantly, contradictory findings were explicitly noted.

### 2.7. Ethical Statement

Ethical approval was not required, as this study used only previously published data and did not involve direct interactions with human or animal subjects.

## 3. Results

### 3.1. Study Selection/Data Selection

Figure 1 shows the PRISMA-ScR flow diagram for study selection. Of 583 database records identified, 15 were removed before screening (one non-English and 14 duplicates). The remaining 568 records were screened by title and abstract. Of these, 422 were excluded (17 review articles, 340 not full text or unrelated to wound healing, 20 conference reports or insufficient methods, and 45 outside 2015–2025). We sought to retrieve 146 reports, but 125 could not be obtained (47 and 78 by reason category). Twenty-one full-text reports were assessed for eligibility, with seven excluded for irrelevant topics, leaving 14 database studies. Additional methods identified three records (one by hand searching and two by citation searching). After removing one duplicate, one hand-searched study was included, resulting in a total of 15 included studies (Table 1).

### 3.2. Characteristics of the Included Studies

Table 1 summarizes the characteristics of the 15 included studies. All the examined potato-derived preparations were from the peel and/or tuber. While 7 of these studies specified the potato variety used (Granola, Tumbay, Dieng, Jayoung, or local cultivars); 8 referred only to *Solanum tuberosum* L. Most of the studies focused on the anti-inflammatory and regenerative (wound-healing) effects of potato-derived metabolites, and several also investigated their antioxidant and antibacterial properties.

Of the 15 studies, 11 used animal models (rats or mice), 3 performed in vitro studies (using RAW264.7 macrophages, IEC-6 intestinal epithelial cells, and THP-1 cells), and 2 included clinical or human skin investigations (minor burn dressing series [15] or human skin safety testing of potato-derived exosomes (SDEs) [28]. Lee et al., [28] for example, combined in vitro and human skin endpoints, whereas Generalov et al. [29] reported both in vivo edema and in vitro THP-1 models. Wound and inflammatory models included oral or gingival mucosa (*n* = 3), cutaneous excision wounds (*n* = 2), burn wounds (*n* = 2), gastric or peptic ulcer models (*n* = 3), general inflammatory edema models (*n* = 3), and macrophage or intestinal epithelial inflammation models (*n* = 3). The intervention formats included ethanol extracts (the most common), aqueous extracts, boiled peel dressings, polysaccharide isolates, peptide fractions, anthocyanin mixtures, and SDEs. Regarding the chemical evidence level, 9 studies used crude extracts with phytochemical screening only, 4 used defined fractions or isolates with partial characterization [29,30,31,32], 1 employed exosomal delivery with molecular profiling [28], and 1 reported a clinical dressing without standardized extract characterization [15]. Notably, the range of experimental models, wound locations, and injury types provided valuable insights into the inflammatory and regenerative phases of wound healing. Combined macroscopic, histopathological, and molecular observations enhanced our understanding of the therapeutic potential of potato-derived metabolites. However, research specifically addressing wound-healing biomarkers remains limited.

**Table 1:** Characteristics of the studies included in this review.

No.	Reference	Variety/Plant Part	Experimental Model	Formulation/Dose	Extraction	Key Findings	Chemical Evidence Level
1	Rosa et al. [33]	Dieng yellow potato/tuber	Cutaneous excision, mice	Gel	Ethanol 96%	Increased fibroblasts, angiogenesis, epithelial thickness	Crude extract
2	Ahmad et al. [34]	Unspecified/tuber	Gastric ulcers, rats	Extract	Ethanol 96%; aqueous	Reduced ulcer size; accelerated healing	Crude extract
3	Santoso et al. [17]	Granola/peel	Palatal oral wounds, rats	Gel 4–6%	Ethanol 96%	Increased fibroblast and collagen; biphasic inflammation	Crude extract
4	Rosas-Cruz et al. [35]	Tumbay yellow/tuber	Dorsal excision, mice	Ointment	Ethanol 96%	Accelerated wound closure	Crude extract
5	Tiarasanti et al. [16]	Granola/peel	Gingival wounds, rats	Gel 4–6%	Ethanol 96%	Faster macroscopic wound closure	Crude extract
6	Silva-Correa et al. [36]	Tumbay/tuber	Second-degree burns, mice	Ointment	Ethanol 96%	Accelerated healing; collagen and fibroblast increases	Crude extract
7	Khadar et al. [37]	Unspecified/tuber	Gastric ulcers; edemas, rats	Extract	Ethanol 96%; aqueous	Reduced ulcer and inflammation	Crude extract
8	Sufiawati et al. [3]	Granola/peel	Palatal oral ulcers, rats	Gel 4–6%	Ethanol 96%	Reduced MMP-9 vs. that after triamcinolone treatment	Crude extract
9	Subrahmanyam. [15]	Unspecified/peel	Human minor burns	Boiled peel dressing	Boiled water	Granulation and epithelialization	Clinical dressing
10	Generalov et al. [30]	Unspecified/peel	Chronic peptic ulcers, rats	STPs (systemic)	Biological isolation	Epithelial regeneration; cytokine modulation	Defined polysaccharide
11	Lee et al. [31]	Jayoung/tuber epidermis	Lipopolisakarida (LPS)-stimulated RAW264.7	CAJY isolate	Anthocyanin isolation	NF-κB/STAT1/3 suppression; reduced iNOS, COX-2, cytokines	Defined anthocyanin mixture
12	Ahmad et al. [38]	Unspecified/tuber	Carrageenan/histamine/formalin edemas, rats	Extract	Ethanol 95%; aqueous	Significant anti-inflammatory activity	Crude extract
13	Lee et al. [28]	Unspecified/exosomes	Human skin safety; keratinocytes	SDEs	Juicing; differential centrifugation + ultracentrifugation	MMP and cytokine suppression; barrier gene induction	Exosomal delivery (EV/delivery system)
14	Generalov et al. [29]	Unspecified/polysaccharide	THP-1; edema models	STPs	Polysaccharide isolation	Cytokine and NFKB1 modulation; NRF2 induction	Defined polysaccharide
15	Basilicata et al. [32]	Unspecified/dehydrated tuber	IEC-6 oxidative-inflammatory stress	Peptide fraction	Simulated digestion	Reduced TNF, COX-2, iNOS; accelerated repair	Defined peptide fraction

### 3.3. Phytochemical Profile and Biological Activities

Flavonoids, polyphenols, tannins, alkaloids, and triterpenoids have consistently been detected in crude potato extracts [16,17,33,35,37,38,39], and anthocyanins are present in both colored and yellow potato preparations [31,33]. Potato polysaccharides (STPs) have been studied as isolated anti-inflammatory and ulcer-healing agents [29,30]. Additionally, bioaccessible peptides (such as patatin and tuberinin) were identified after the simulated gastrointestinal digestion of dehydrated potatoes [32], and SDEs were characterized as nanovesicles with anti-inflammatory and matrix-modulating properties [28]. Reported metabolite concentrations vary substantially among potato varieties, plant parts, extraction methods, and harvest conditions, thereby limiting direct comparability across studies.

Table 2 summarizes reported biological activities and their mechanisms. Among the 15 studies included here, 6 predominantly or exclusively reported regenerative activity, 3 only described anti-inflammatory activities, and 6 reported both anti-inflammatory and regenerative effects. Only six studies reported explicit inflammatory or regenerative biomarkers (MMP-9, TNF-α, IL-6, IL-1β, IFN-γ, COX-2, PGE_2_, NF-κB, STAT1/3, IL-4, and IL-10), underscoring the predominance of histopathological over molecular endpoints. Overall, the types of chemical metabolites examined were similar, but the reported concentrations may have differed due to various factors, including extraction methods, potato varieties, and the planting and harvesting periods of the studied potatoes. Flavonoids, polyphenols, and alkaloids have almost always been identified, and researchers have not thoroughly examined variations in the contents of other chemical metabolites. The majority of the studies fulfilling the inclusion criteria were in vivo studies that reported the effects of potato peel and tuber extracts both macroscopically and microscopically, including ulcer size changes and the involvement of cells (such as inflammatory cells) in the wound-healing process. Other inflammatory biomarkers have not been extensively studied or fall outside the inclusion criteria of this review; therefore, they were not included in the data extraction. Comprehensive research on biomarkers in the different phases of wound healing would greatly aid in the development of potato extract as a standardized herbal therapy.

Based on the extraction of data from the 15 selected articles, there were several new scientific findings from in-depth research on the molecular activity of potato extract, namely: (1) Granola potato peel ethanol extract (GPPEE) 4% significantly suppresses the expression of MMP-9, more effectively than the standard corticosteroid triamcinolone acetonide 0.1% in mucosal wounds. The suppression of MMP-9 is crucial to prevent excessive collagen degradation during the inflammatory phase [3]; (2) potato extracts may have a mixture of p-coumaroyl anthocyanins (petanin, peonanin, malvanin, and/or pelanin [CAJY]) that specifically inhibit the STAT1 and STAT3 signaling pathways and inactivate NF-κB in the inflammation phase [31]; (3) STPs reduce proinflammatory cytokines (IL-1β and IFN-γ) and simultaneously increase anti-inflammatory cytokines (IL-4) [30].

Other scientific information indicates that potato extract can work through multi-target mechanisms in modulating inflammation, namely: (1) tuber extract significantly reduces edema induced by carrageenan, histamine, and formalin by inhibiting vascular permeability and the release of prostaglandins [34,37,38]; (2) intravenous STPs triggers differentiated gastric mucosa regeneration without the formation of coarse scars, which is an advantage compared to the effects of comparator drugs such as omeprazole [30]; (3) the anthocyanins and flavonoids in potatoes inhibit the COX-2 and iNOS pathways and reduce the production of reactive oxygen species (ROS) [31].

### 3.4. Thematic Findings by Research Question

#### 3.4.1. Bioactive Metabolites

Five major bioactive metabolite classes emerged from this synthesis: (1) flavonoids and anthocyanins, (2) polyphenols/phenolic acids, (3) alkaloids and glycoalkaloids, (4) polysaccharides, and (5) peptides/proteins. In addition, potato-derived exosomal vesicles (SDEs) were mapped as extracellular vesicles/delivery systems that transport bioactive cargo, rather than as a sixth metabolite class [28]. Peel preparations were enriched in phenolics and flavonoids, whereas tuber extracts frequently contained tannins, sterols, and alkaloids. Notably, none of the included studies compared the peels and tubers using identical extraction and wound models.

#### 3.4.2. Experimental Models and Regeneration

In wound-healing studies, potato-derived preparations have consistently been associated with regenerative and anti-inflammatory benefits. Strong convergence was observed for increased fibroblast proliferation (*n* = 5) [17,30,33,36], enhanced collagen deposition (*n* = 4) [17,30,33,36], and accelerated wound closure or reduced ulcer area (*n* = 8) [16,28,30,33,34,35,36,37]. Moderate evidence supported improved epithelial thickness and reepithelialization (*n* = 4) [15,17,30,33], and angiogenesis/neovascularization (*n* = 3) [17,30,33], although angiogenesis was not significant in the oral mucosa model [17]. Three gastric and peptic ulcer models were developed for mucosal tissue repair [30,34,37] and four studies used systemic inflammation or in vitro models without cutaneous or oral mucosal wound endpoints [29,31,32,38].

#### 3.4.3. Molecular Pathways and Biomarkers

Table 3 presents the mapping of molecular targets. Only 6 of the 15 studies reported explicit molecular biomarkers.

#### 3.4.4. Mechanistic Findings

Three mechanistically informative findings were identified in the 15 articles. First, MMP-9 modulation in oral mucosal wounds: 4% and 6% GPPEE gel reduce MMP-9 expression more effectively than 0.1% triamcinolone acetonide, with 4% GPPEE treatment showing the lowest mean expression by day 14 [3]. These data suggest an ECM-modulating effect during the inflammatory–proliferative transition phase. Second, inhibitory effects of CAJY: peonanin, malvanin, and pelanin from Jayoung potato epidermis inhibit LPS-induced ROS, iNOS, COX-2, TNF-α, and IL-6 in RAW264.7 macrophages via NF-κB and STAT1/3 inactivation [31]. Third, STPs’ effects: STPs reduce proinflammatory cytokines (IL-1β, IFN-γ, and TNF) and increase IL-4 in peptic ulcer models, with dose-dependent ulcer index reductions comparable to or exceeding those of omeprazole and ranitidine. In THP-1 cells, STPs downregulates IL-6, TNF-α, IL-1β, and NFKB1, and upregulates IL-10 and NRF2 [29].

### 3.5. Summary of Reported Activities

According to the information given in Section 3.3 and Table 2, six studies reported predominantly regenerative activity, three identified anti-inflammatory activities, and six reported both anti-inflammatory and regenerative effects of the different potato-derived metabolites isolated and tested. When considered by study design, three employed molecular inflammation models without direct wound endpoints and two provided clinical dressing or human skin safety evidence; these design categories overlapped with the activity-based classification above. Only six studies reported explicit inflammatory or regenerative biomarkers (MMP-9, TNF-α, IL-6, IL-1β, IFN-γ, COX-2, PGE_2_, NF-κB, STAT1/3, IL-4, and IL-10). As noted in Section 3.3, comprehensive biomarker profiling across the healing phases remains limited.

## 4. Discussion

Potatoes are used in traditional medicine as fever reducers, pain relievers, digestive aids, and wound healers and to treat gastritis and other maladies [4]. This scoping review mapped 15 primary studies (published between 2015 and 2025) that investigated potato-derived bioactive metabolites involved in the modulation of inflammation and tissue regeneration during wound healing. The synthesized evidence supports the multi-target therapeutic profile of *Solanum tuberosum* L. preparations, acting through antioxidant, anti-inflammatory, and proregenerative mechanisms rather than a single molecular pathway. Although most included studies investigated crude potato extracts rather than isolated metabolites, the available phytochemical evidence enabled classification into five major bioactive metabolite classes, with exosomal vesicles treated separately as a delivery system. This classification provides a mechanistic framework for interpreting their biological activities. Nevertheless, this field remains at a promising preclinical stage with a fragmented mechanistic and translational foundation. This discussion interprets the findings relative to each research question, synthesizes novel evidence from the included literature, articulates the original contribution of this scoping review, and addresses the five identified priority research gaps.

In addition to models of wounds applied to the skin, potato has also been viewed as a food that may suppress inflammation. Reddivari et al. [13] proposed that potato types rich in starch that is difficult to digest, fiber, and purple pigments (anthocyanins) may reduce intestinal inflammation and prolonged inflammation throughout the body, in part by affecting gut microbial products and inflammatory pathways, including responses to bacterial toxin (LPS). This view helps explain why anthocyanin- and phenolic-rich potato preparations often affect the NF-κB and COX-2 pathways and inflammatory mediators (cytokines) in macrophage and epithelial cells in this review. However, gut-centered evidence should not be taken as direct proof that potato heals skin wounds or oral ulcers. In the wound-related studies in this review, anti-inflammatory claims are strongest when supported by tissue improvement under the microscope or clear molecular markers. Food-based effects mainly provide mechanistic clues for further research, not direct proof that wounds close.

The first stage of wound healing is inflammation, which is characterized by pain, swelling, and the release of proinflammatory mediators. When inflammation resolves, healing occurs through cell regeneration, which closes both ulcers and other wounds and restores normal conditions. Research on the chemical metabolites in potato skin and tubers has identified phenolics, flavonoids, and anthocyanins, which are more abundant in the peel. Specifically, according to published studies, potato peel contains polyphenols, flavonoids, anthocyanins, and other compounds, whereas potato tubers contain tannins, flavonoids, triterpenes, and alkaloids [8,18]. In addition, potato extracts contain minerals, such as fiber, vitamin C, folic acid, calcium (Ca), iron (Fe), potassium (K), and sodium (Na) [8]. However, the composition of these metabolites in potato peels and tubers varies with a variety of factors, such as potato variety, extraction method, and harvest time [9,10,19,20,21,22].

### 4.1. Interpretation of Metabolite Bioactive and Wound-Healing Phases

**Figure 2:**
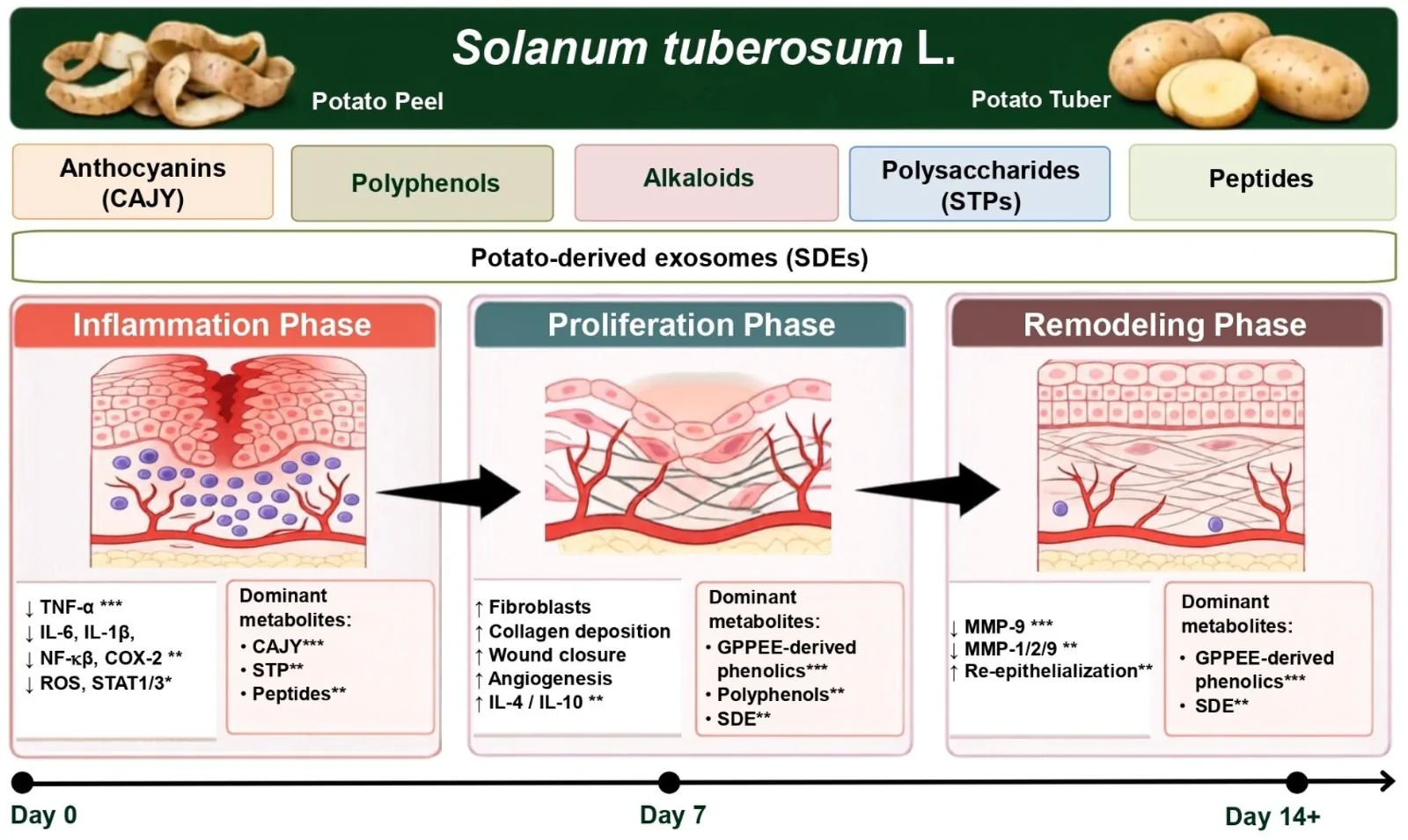
Conceptual framework summarizing the reported associations between potato-derived bioactive metabolites, molecular targets, and the three phases of wound healing (inflammation, proliferation, and remodeling). Arrows represent associations reported in the included studies and do not imply direct causal relationships. Upward arrows (↑) indicate increased expression or biological activity, whereas downward arrows (↓) indicate decreased expression or biological activity. Evidence strength is categorized as *** strong, ** moderate, and * weak. CAJY, *p*-coumaroyl anthocyanin mixture from the ‘Jayoung’ potato cultivar; STPs, *Solanum tuberosum* L. polysaccharides; SDEs, *Solanum*-derived extracellular vesicles (a non-metabolite delivery system); GPPEE, Granola potato peel ethanol extract.

Potato (*Solanum tuberosum* L.) extract contains various bioactive metabolites: flavonoids, polyphenols (like chlorogenic acid), alkaloids (α-chaconine and α-solanine), and vitamins and minerals (vitamin C, vitamin B6, and zinc [Zn]) [3,10,17,18], all of which accelerate wound healing [12]. Potato extract metabolites appear to act across multiple phases of wound healing (Figure 2). During the inflammatory phase, flavonoids, anthocyanins, and polysaccharides may reduce proinflammatory cytokines (TNF-α, IL-6, IL-1β, and IFN-γ), inhibit COX-2/iNOS signaling, and modulate NF-κB and STAT pathways [3,29,30,31,32]. During proliferation, phenolic compounds and anthocyanins may stimulate fibroblast activity, collagen synthesis, angiogenesis, and reepithelialization [16,17,33,35,36]. During remodeling, MMP-9 suppression by GPPEE gel suggests protection against excessive ECM degradation [3].

The phase-biomarker-metabolite integration framework linking *Solanum tuberosum* L. bioactive metabolites to wound-healing stages, synthesized from selected and supported articles, is shown in Figure 2. Such a framework integrates the five potato bioactive metabolite classes from the peel or tuber and exosomal vesicles as a delivery platform across the three overlapping wound-healing phases. Inflammation, proliferation, and remodeling phases were aligned with reported molecular biomarkers and dominant metabolite classes. Molecular biomarker grades were assigned as follows (see Table 3): strong (≥3 studies), moderate (2 studies), and weak (a single study or in vitro studies only). Histopathological endpoints (fibroblast proliferation, collagen deposition, and wound closure) are reported separately without molecular evidence grades. In the inflammatory phase, an ulcerated wound shows increased levels of proinflammatory cytokines (IL-1β, TNF-α, IL-6, IFN-γ, COX-2, and NF-κB), increased oxidative stress, increased MMP activity, and tissue damage. Metabolites in potato extracts reduce proinflammatory cytokine expression, inhibit inflammatory pathways, and decrease oxidative stress [3,30]. CAJY, STPs, and peptides are associated with reduced IL-1β, TNF-α, IL-6, IFN-γ, COX-2, NF-κB, and ROS, reflecting reported convergent anti-inflammatory molecular signaling in included primary studies, with graded evidence certainty per Table 3 herein. As antioxidant and antibacterial agents, CAJY, STPs, and peptides reduce the levels of free radicals and bacteria that cause oxidative stress, hinder healing, and promote chronic inflammation [40]. These metabolites accelerate wound closure and enhance ulcer healing during the regeneration phase by promoting fibroblast development, collagen synthesis, granulation tissue creation, angiogenesis, and reepithelialization. Remodeling connects GPPEE and SDEs with MMP-9/MMP-1/2 attenuation and reepithelialization.

While the predominance of crude extract studies limits causal attribution, defined preparations, such as CAJY [31], STPs [29,30], gastrointestinal bioaccessible peptides [32], and SDEs [28], provide strong chemical evidence. However, substantial pathway-level data is only available for STPs and CAJY, and neither of these compounds has been tested in a standardized comparative wound-healing trial against clinical standards.

### 4.2. Flavonoids and Anthocyanins

The potential anti-inflammatory role of the metabolites in potato extracts can be observed through several identified agents. Flavonoids and anthocyanins are the most frequently reported metabolites in peel and colored tuber preparations. Moreover, published phytochemical tests have shown that flavonoids are almost always present in potato extracts. These metabolites can act as antioxidants, anticarcinogens, antibacterial agents, anti-inflammatory agents, antiallergens, and analgesics. These compounds may exert antioxidant and anti-inflammatory effects by modulating the COX/LOX pathway, reducing leukocyte infiltration, and suppressing NF-κB, MAPK, and Akt signaling [31,41,42,43,44,45]. Lee et al. [31] provided the strongest compound-level evidence, demonstrating that CAJY attenuates LPS-induced ROS and inflammatory mediators via NF-κB and STAT1/3 inactivation. The increase in free radicals is due to a decrease in antioxidants that neutralize ROS. When free radicals exceed antioxidant levels, oxidative stress occurs, hindering the healing process and causing chronic inflammation [44].

In addition, flavonoids exert anti-inflammatory effects by reducing the expression of TNF-α, IL-1, and COX-2. TNF-α is a crucial cytokine in the inflammatory phase [41,42,43,44]. Flavonoids and anthocyanins in potato extracts act as anti-inflammatory agents by inhibiting the cyclooxygenase and lipoxygenase pathways and reducing histamine levels and leukocyte accumulation, thereby alleviating inflammation. Al-Khayri et al. [45] claimed that flavonoids lower inflammation and oxidative stress by targeting the NF-κB, MAPK, and Akt signaling pathways [31]. Interestingly, both anthocyanins and flavonoids have been shown to reduce proinflammatory cytokines, including TNF-α, IL-6, IL-8, IL-1β, IL-17, and IFN-γ [34,45,46]. Flavonoids also inhibit MMPs, which are enzymes that break down collagen; consequently, flavonoids increase the rate and amount of collagen available for forming a new wound matrix, accelerating wound healing [17]. Anthocyanins mitigate collagen damage by inhibiting cell damage, increasing vascularization, initiating DNA synthesis, and promoting fibronectin and fibroblast synthesis, potentially contributing to wound healing [33,47].

Rosa et al. [33] partly attributed the effects of yellow potato gel on fibroblasts, angiogenesis, and epithelial thickness to anthocyanins (15–40 mg/100 g). However, this inference was not confirmed by in vivo testing of isolated compounds. Chlorogenic acid and other phenolic acids, which are abundant in potato peel, have been discussed as potential contributors to antioxidant and MMP-modulating activities [10,36]; however, chlorogenic acid has not been tested in any wound model. Evidence grade: moderate for anti-inflammatory signaling; weak for direct wound-healing attribution to specific flavonoids/anthocyanins in vivo.

### 4.3. Polyphenols and Phenolic Acids

Potato peels and tubers are rich in phenolic metabolites, and higher concentrations are often reported in the peel than in the flesh [9,10]. Polyphenols can reduce oxidative stress, support collagen matrix formation, and inhibit inflammatory enzyme cascades [15,30]. Rosas-Cruz et al. [35] and Silva-Correa et al. [36] showed that phenolic-rich Tumbay potato ointments accelerate closure and histological repair in in vivo excision and burn models, although phenolic quantification varied between the peel and pulp fractions. Phenolic acids are directly linked to human health; they are rich in antioxidants, help protect against chronic diseases, maintain blood pressure, and protect against microorganisms. Compared with vegetables such as tomatoes, onions, and carrots, potatoes have the highest levels of phenolic metabolites, generally 23.55–48.39 mg, which are present in the skin and flesh [4,48]. Therefore, potatoes are a good source of phenolic metabolites, with higher total phenolic contents than other fruits and vegetables. Though present in both potato peels and tubers, the peel has the highest concentrations of phenolic metabolites [10].

Polyphenol metabolites in potato extract have anti-inflammatory activity; they regulate cellular activity in inflammatory cells, enzyme activity involved in arachidonic acid metabolism (phospholipase and COX), and arginine metabolism, and modulate the production of proinflammatory molecules [31,40]. Lee et al. [31] reported that potato-derived metabolites inhibit iNOS and COX-2. This inhibition reduces PGE2 production, thereby reducing pain and inflammation [31]. During wound healing, the regeneration phase follows the inflammatory phase; this regeneration phase is marked by epithelial cell closure of the wound. As shown in Table 1, all tested potato extracts affect the wound-healing regeneration process, both clinically and histopathologically. Evidence grade: moderate for histological improvement; weak for isolated phenolic acid mechanisms in wound models.

### 4.4. Polysaccharides, Peptides, and Novel Delivery Systems

Generalov et al. [29,30] advanced the understanding of STPs as an immunomodulatory agent that rebalances pro- and anti-inflammatory cytokines and promotes differentiated mucosal regeneration without coarse scarring. The effects of STPs are comparable to those of ibuprofen in edema models and omeprazole/ranitidine in chronic peptic ulcer healing [29,30]. These findings extend potato research beyond topical wound models to include mucosal regeneration with cytokine-level evidence.

Basilicata et al. [32] identified patatin- and tuberinin-derived peptides that reduce TNF-α, COX-2, and iNOS and enhance wound repair in IEC-6 cells under inflammatory stress. Furthermore, Lee et al. [28] reported that SDEs suppress MMP-1, MMP-2, MMP-9, and inflammatory cytokines while promoting keratinocyte wound closure and skin barrier gene expression, with favorable human skin safety profiles. These studies suggest the emergence of delivery platforms beyond conventional gels and ointments. Evidence grade: moderate for polysaccharide cytokine modulation; weak-to-moderate for peptides and exosomal delivery systems in vivo wound healing.

### 4.5. Minerals and Vitamins

The mineral content of potatoes includes Ca, phosphorus (P), Fe, magnesium (Mg), K, Na, sulfur (S), copper (Cu), manganese (Mn), and cobalt (Co). Research reports have mentioned other components of potatoes besides their mineral content, including calories, fiber, vitamin C, and folic acid. Mineral and vitamin metabolites in potato extract play important roles in cells and tissues. Vitamin C, for example, is an antioxidant that helps maintain skin health and wound healing by increasing fibroblast production, thereby accelerating the wound-healing process [49]. Notably, potatoes contain approximately 20 mg of vitamin C per 100 g [12].

Data from all studies indicated that potato extracts accelerate wound healing, both clinically and histopathologically. These results warrant further research on the effects of the extract on active biomarkers during the inflammatory, proliferative, and regenerative phases of wound healing. Comprehensive results can be obtained before conducting human clinical trials. These findings support the translational potential of potato-derived bioactive metabolites for the treatment of inflammation-related mucosal disorders.

Taken together, these results indicate that the bioactive metabolites derived from *Solanum tuberosum* L. have multiple effects on wound healing. These metabolites simultaneously modulate inflammatory signaling (such as NF-κB and COX-2), oxidative stress, and extracellular matrix remodeling, rather than single pathways. This multi-mechanistic action may account for their consistent effectiveness in various experimental models.

### 4.6. Research Gaps, Limitations, and Future Directions

We found the following research gaps:

(1) Standardization of extracts and formulations, with emphasis on dose normalization, marker compounds, and batch reproducibility. Among the 15 included studies, nine relied on crude extracts with phytochemical screening only, whereas only a minority used chemically defined fractions or isolates (e.g., CAJY, STPs, and peptide fractions) or characterized delivery systems (SDEs) [28,29,30,31,32]. Although most studies have been conducted in vivo, their key limitations include the lack of standardized extraction methods, formulations, and solvents, as well as the use of different plant varieties and plant parts, leading to inconsistent metabolite profiles and the study of only a limited number of biomarkers. No head-to-head comparisons of peel and tuber contents using identical protocols were found. These factors hinder comparability across studies.

To enhance the reproducibility and comparability of future studies, extract standardization should be prioritized. Researchers should report doses in pharmacologically relevant units rather than relying solely on nominal gel or ointment concentrations to enable meaningful comparisons across experimental models. Standardized marker compounds should be quantified according to the preparation type, such as chlorogenic acid and related phenolics for peel-derived extracts, total anthocyanins for colored cultivars, and polysaccharide content for STPs preparations. Comprehensive documentation of potato cultivar, plant part, harvest stage, and extraction conditions, together with chromatographic profiling of marker compounds where appropriate, would further improve batch-to-batch reproducibility and strengthen the reliability and clinical relevance of potato-derived wound-healing products.

Future research should prioritize standardized extraction protocols, improved phytochemical characterization, broader biomarker profiling, dose–response studies, and well-designed clinical trials to strengthen the evidence base for potato-derived wound-healing products.

(2) Fragmented molecular biomarker evidence. Only six studies have explicitly reported molecular biomarkers regarding potato-derived metabolite effects. Most studies relied on fibroblast count, collagen deposition, or wound area changes without pathway validation. The following minimal biomarker panel is proposed for future studies: MMP-9, TNF-α, IL-6, IL-1β, COX-2, NF-κB, collagen deposition, and fibroblast proliferation, assessed at defined time points across inflammatory and proliferative phases.

(3) Compound-specific causality. Solanine, chaconine, chlorogenic acid, and individual anthocyanins have not yet been tested as isolated agents in in vivo wound models. Bioassay-guided fractionation using standardized oral or cutaneous wound models is needed to move beyond phytochemical screening of crude extracts.

(4) Clinical and translational evidence. Despite promising preclinical data, including GPPEE effects comparable to those of triamcinolone [17], high effectiveness of 2% ointment against burns [36], and superior MMP-9 suppression compared to that of corticosteroids [3], no RCT of standardized potato extract versus clinical standards exists. Reported safety data (LD50 > 2000 mg/kg for tuber extracts [38], IC50 235.80 μg/mL for GPPEE [17], and topical SDEs safety [28]), support further development of potato-derived metabolite-based therapies but do not substitute for efficacy trials. The priority clinical indications identified include traumatic oral mucosal ulcers, where histopathological and molecular evidence is most concentrated.

(5) Multidisciplinary and complex wound models. None of the included studies applied network pharmacology, metabolomics, or transcriptomic integration across the datasets. Complex models (including diabetic, infected, or chronic wound models) were absent. Short-term priorities include omics-guided target prediction, whereas mid-term priorities include diabetic wound and infected wound models with standardized peel extract. In addition, long-term priorities include RCTs and pharmacokinetic/safety evaluations of glycoalkaloids in repeated topical use.

This review was limited by several factors: a bias assessment was not conducted for some articles, consistent with the nature of scoping reviews. In addition, some studies lacked clear descriptions of their blinding methods (whether single- or double-blind), and many did not specify the maturity or variety of potatoes used, which is important because different varieties may present different chemical profiles. Finally, mechanistic claims from the in vitro inflammation models were cautiously extrapolated to wound healing.

Another limitation is the lack of comprehensive in vivo research publications on potato extracts, including pharmacokinetics (absorption, metabolism, and toxicity), dosage, and their relationship to safety profiles. Comprehensive pharmacological and clinical studies evaluating the bioavailability, toxicity, optimal dosage, and safety profiles are crucial for therapeutic applications in humans. The literature on the anti-inflammatory and regenerative activities of potato peel extract, particularly with respect to indicators such as fibroblast activity, collagen deposition, new blood vessel formation, and reduced inflammatory cell numbers, remains limited.

Despite these limitations, this review provides the first structured evidence map linking potato bioactive metabolite classes and delivery systems, molecular targets, wound models, and translational gaps covering the literature from 2015 to 2025. Future research should establish reference standards for variety, extraction, formulation, and dosing, particularly for GPPEE (4–6%), which showed consistent oral mucosal benefits [3,16,17].

The advantages and potential of potato extract as an herbal medicine for wound-healing therapy are very promising. For example, extracts are obtained from potato residues, thereby reducing organic waste. In addition, analyses showed that potato-based preparations (gel/ointment 2–6%) offer a competitive therapeutic profile. Their main advantage is their multi-target capability (simultaneous anti-inflammatory, antioxidant, and antibacterial properties), which single drugs such as TCA do not possess. Additionally, potatoes have a high safety profile with an LD50 > 2000 mg/kg, avoiding corticosteroid side effects, such as dermal thinning or the risk of oral candidiasis. Moreover, potatoes are abundant, easy to find and process, and cheap, and constitute an effective raw material for developing countries. Moreover, freshly sliced potatoes have long been used in traditional medicine as an anti-inflammatory remedy [3,34].

### 4.7. Translational and Clinical Implications

Potato peel valorization aligns with sustainable pharmacy and waste-reduction goals, particularly when the peel is discarded during food processing [16,17,18]. Topical gels and ointments (2–6%) are feasible for mucosal and cutaneous applications, with multi-target activity (anti-inflammatory, antioxidant, and pro-regenerative) offering potential advantages over single-mechanism agents such as corticosteroids [3,17].

Translational development should proceed in the following stages: (1) standardizing extract composition and stability, (2) validating biomarker panels in preclinical models, (3) conducting dose–response and toxicity studies for glycoalkaloids, and (4) initiating pilot clinical studies in oral mucosal wounds before expanding to cutaneous and burn indications. Recommendations herein are preclinical-informed and should not be interpreted as clinical practice guidelines.

### 4.8. Synthesis and Original Contribution

The synthesis of the 15 included studies revealed findings that were not previously integrated into a single potato-based wound healing review. Three linked studies establish a progressively deeper oral mucosal healing evidence chain for GPPEE: macroscopic gingival closure [16], histopathological palatal regeneration with biphasic inflammation [17], and MMP-9 suppression superior to that of triamcinolone [3], with an outcome-dependent optimum (6% for early closure; 4% for late MMP-9 suppression). At the component level, Lee et al. [31] demonstrated dual NF-κB/STAT1/3 inhibition by defined anthocyanins (CAJY), while Generalov et al. [29,30] demonstrated STP-mediated cytokine rebalancing and differentiated mucosal regeneration. Emerging therapeutic modalities include the use of bioaccessible peptides [32] and SDEs [28]. Subrahmanyam [15] provided the only human burn wound healing study. The study suggests that, in direct contact with burn wounds, potato peels act as moist barriers rather than antimicrobial agents.

Methodologically, this review contributes to the first structured PRISMA-ScR evidence map (2015–2025) by linking bioactive metabolite classes and delivery systems, molecular targets, models, and translational gaps. Descriptive evidence grades (Table 3) and explicit mapping, integrative framework (metabolite → target → healing phase) construction, and GPPEE (4–6%) identification as the most promising preclinical development candidate could emerge only through cross-study synthesis of relevant literature. The structured five-gap agenda (standardization, biomarkers, compound validation, clinical translation, and omics/complex models) establish a foundation for future research.

### 4.9. Comparison with Prior Reviews

To demonstrate the originality of the present scoping review relative to recent potato-focused literature, Table 4 compares representative prior reviews with the current work across scope, wound-healing mapping, molecular pathway coverage, preclinical/clinical evidence, and translational outputs.

As summarized in Table 4, previous reviews have primarily addressed phytochemical composition, antioxidant capacity, nutritional value, or general pharmacology of potato metabolites [4,7,8,10,11,12,13,18]. The present PRISMA-ScR review provides the first structured evidence map that links potato-derived bioactive metabolite classes and delivery systems to wound-healing phases, molecular targets, and translational gaps. It also grades mechanistic certainty (Table 3), including the oral mucosal GPPEE evidence chain [3,16,17], STP-mediated mucosal regeneration [29,30], SDEs nanovesicle platforms [28] and proposes an actionable research agenda for standardization and clinical development. These features collectively distinguish the present work from earlier potato reviews and support its originality in the wound-healing domain.

## 5. Conclusions

Potato extracts from the skin or tubers are rich in metabolites that accelerate healing from inflammation to regeneration. Flavonoids, phenols, and alkaloids have been widely tested for their antioxidant, anti-inflammatory, and regenerative effects on wound healing. The diversity of potato types and metabolites offers opportunities to identify superior varieties with high metabolite contents and pharmacological activities prior to human clinical trials. We determined the bioactive metabolites in potato peels and tubers that reduce inflammation and promote regeneration of mucosal wounds.

## Figures and Tables

**Table 2:** Biological activities and mechanisms of potato-derived preparations (metabolites and delivery systems).

No.	Study	Extraction Method	Bioactive Constituents or Delivery System	Inflammation	Regeneration	Reported Biomarkers
1	Rosa et al. [33]	Maceration	Anthocyanins	Not reported	↑ Fibroblasts, angiogenesis, epithelial thickness	None
2	Ahmad et al. [34]	Maceration	Tannins, flavonoids, triterpenes, alkaloids	Not reported	↓ Ulcer size; accelerated healing	None
3	Santoso et al. [17]	Maceration	Phenols, flavonoids, saponins, tannins, triterpenes, alkaloids	↓ Inflammatory infiltration (days 7–14)	↑ Fibroblasts, collagen, vascularization	None
4	Rosas-Cruz et al. [35]	Maceration	Gallic acid (phenol)	Not reported	↑ Fibroblasts, collagen	None
5	Tiarasanti et al. [16]	Maceration	Tannins, saponins, triterpenes, flavonoids, alkaloids	Not reported	Accelerated macroscopic closure	None
6	Silva-Correa et al. [36]	Maceration	Not specified	Not reported	↑ Collagen, fibroblasts	None
7	Khadar et al. [37]	Soxhlet	Tannins, sterols, flavonoids, triterpenes, alkaloids	↓ Inflammation and ulcer severity	Not reported	None
8	Sufiawati et al. [3]	Maceration	Tannins, saponins, triterpenes, flavonoids, alkaloids	↓ Inflammatory response	Accelerated healing	↓ MMP-9
9	Subrahmanyam [15]	Boiled peel	Polyphenols	Not reported	Rapid epithelialization and closure	None
10	Generalov et al. [30]	Biological isolation	STPs	↓ Inflammation; ulcer healing	Differentiated mucosa; ↑ vessels, fibroblasts	↓ IL-1β, IFN-γ; ↑ IL-4
11	Lee et al. [31]	Maceration	p-coumaroyl anthocyanins (CAJY)	↓ iNOS, COX-2, TNF-α, IL-6, ROS	Not reported	NF-κB, STAT1/3 suppression
12	Ahmad et al. [38]	Maceration	Tannins, flavonoids, alkaloids, sterols, triterpenes	↓ Edema	Regenerative support	None
13	Lee et al. [28]	Centrifugation	SDEs (extracellular vesicles/delivery system)	↓ Inflammation	Accelerated keratinocyte closure	↓ MMP-1/2/9; ↓ TNF, IL-6
14	Generalov et al. [29]	Polysaccharide isolation	STPs	↓ Edema and inflammation	Not reported	↓ IL-6, TNF-α, IL-1β, NFKB1; ↑ IL-10; ↑ NRF2
15	Basilicata et al. [32]	Dehydration/digestion	Patatin, tuberinin peptides	↓ Inflammation	↑ Cell migration/repair	↓ TNF-α, COX-2, iNOS

Note: ↓ = Decreasing, ↑ = Increasing.

**Table 3:** Mapping of molecular targets in the studies included in this review.

Pathway/Biomarker	Supporting Studies	Evidence Grade	Main Metabolite Association
NF-κB inhibition	Lee et al., 2017 [31]; Generalov et al., 2025 [29]	Moderate	Anthocyanins; polysaccharides
STAT1/STAT3 suppression	Lee et al., 2017 [31]	Weak	CAJY
COX-2/iNOS/PGE_2_ reduction	Lee et al., 2017 [31]; Basilicata et al., 2019 [32]	Moderate	Anthocyanins; potato peptides
TNF-α reduction	Lee et al., 2017 [31]; Basilicata et al., 2019 [32]; Lee et al., 2025 [28]; Generalov et al., 2025 [29,30]	Strong	Multiple classes
IL-6, IL-1β, IFN-γ modulation	Generalov et al., 2025 [29,30]	Moderate	STPs
IL-4/IL-10 upregulation	Generalov et al., 2025 [29,30]	Moderate	STPs
MMP-9 suppression	Sufiawati et al., 2025 [3]	Weak	GPPEE
MMP-1/2/9 suppression	Lee et al., 2025 [28]	Weak	SDEs (delivery system)
NRF2/antioxidant response	Generalov et al., 2025 [29]	Weak	STPs

**Table 4:** Comparison of the present scoping review with recent reviews on *Solanum tuberosum* L. bioactive metabolites.

Review (Year)	Primary Focus	Wound-Healing Phase Mapping	Molecular Pathways (NF-κB, COX-2, MMPs, Cytokines)	In Vivo/Clinical Wound Evidence	Evidence Grading	Structured Gap/Translational Agenda
Sahani et al., 2023 [4]	Traditional use, phytochemistry, pharmacology, health benefits	No	Limited/nonspecific	Limited	No	No
Das et al., 2017 [7]	Pharmacological properties overview	No	Limited	Limited	No	No
Jimenez-Champi et al., 2023 [18]	Peel bioactive compounds; food-industry applications	No	No	No	No	Limited (valorization)
Sahair et al., 2018 [8]	Botanical, phytochemical, pharmacological, nutritional significance	No	Limited	Limited	No	No
Akyol et al., 2016 [10]	Phenolic compounds in potato and by-products	No	Limited (antioxidant context)	No	No	Limited
Xu et al., 2023 [12]	Potato as functional food	No	Limited (nutrition/inflammation)	No	No	Limited
Al-Weshahy & Rao, 2012 [11]	Peel phytochemical antioxidants/nutraceuticals	No	Limited	No	No	Limited (nutraceutical use)
Reddivari et al., 2019 [13]	Potato as an anti-inflammatory food	No (gut/systemic focus)	Partial (inflammatory signaling)	No (not wound models)	No	Limited
Present scoping review (2015–2025)	Wound healing: inflammation modulation and tissue regeneration	Yes (inflammation, proliferation, remodeling)	Yes (NF-κB, STAT1/3, COX-2/iNOS, cytokines, MMPs; graded in Table 3)	Yes (15 included studies: in vivo, mechanistically relevant in vitro, limited clinical/human skin)	Yes (descriptive evidence grades)	Yes (five-priority agenda: standardization, biomarkers, compound validation, clinical translation, omics/complex models)

## Data Availability

No new data were created or analyzed in this study. Data sharing is not applicable to this article.

## Supplementary Materials

The following supporting information can be downloaded at: https://www.mdpi.com/article/10.3390/biomedicines14081758/s1, Table S1: PRISMA-ScR Checklist; Search Strategy by Database; Table S2: Evidence Grading Criteria.

## Abbreviations

The following abbreviations are used in this manuscript:

AKT	Protein kinase B (PKB)
CAJY	*p*-coumaroyl anthocyanin mixture from Jayoung potato
COX	Cyclooxygenase
ECM	Extracellular matrix
GPPEE	Granola potato peel ethanol extract (gel)
IFN-γ	Interferon gamma
IL-1β	Interleukin 1 beta
IL-6	Interleukin 6
IL-8	Interleukin 8
IL-17	Interleukin 17
iNOS	Inducible nitric oxide synthase
LPS	Lipopolisakarida
MAPK	Mitogen-activated protein kinase
MMP	Matrix metalloproteinase
NF-κB	Nuclear factor kappa B
NRF2	Nuclear factor erythroid 2–related factor 2
PGE_2_	Prostaglandin E_2_
ROS	Reactive oxygen species
TNF-α	Tumor necrosis factor alpha
SDEs	potato-derived exosomes
STAT	Signal transducer and activator of transcription
TCA	Triamcinolone Acetonide
STPs	*Solanum tuberosum* L. polysaccharides

## References

[1] Prayoga D.K., Aulifa D.L., Budiman A., Levita J. (2024). Plants with Anti-Ulcer Activity and Mechanism: A Review of Preclinical and Clinical Studies. Drug. Des. Dev. Ther..

[2] Glick M., Greenberg M.S., Lockhart P.B., Challacomb S.J. (2021). Burket’s Oral Medicine.

[3] Sufiawati I., Finita N., Hidayat W., Yuslianti E.R. (2025). Potato Peel Extract Reduces MMP-9 Expression During Oral Mucosal Wound Healing in Rats. J. Inflamm. Res..

[4] Sahani G., Ghadage P., Raj A.S., Prakash A., Mohammed F. (2023). *Solanum tuberosum* L.: A review on traditional use, phytochemistry, pharmacological aspects, and health benefits. Ann. Phytomed..

[5] Cheng S., Wang H., Pan X., Zhang C., Zhang K., Chen Z., Dong W., Xie A., Qi X. (2022). Dendritic Hydrogels with Robust Inherent Antibacterial Properties for Promoting Bacteria-Infected Wound Healing. ACS Appl. Mater. Interfaces.

[6] Zhu P., Li X., Liu S., Ye S. (2026). A Modular Bioadhesive Incorporating Mussel Adhesive Protein and EGF for Robust Growth Factor Immobilization and Enhanced 4 Wound Healing Chemical Transformations, Tianjin Key Laboratory of Function and Application of Laboratory of Pathobiology, Ministry of Education, Nanomedicine and Translational. Biomacromolecules.

[7] Das K., Krishna P., Sarkar A., Ilangovan S.S., Sen S. (2017). A review on pharmacological properties of Solanum tuberosum. Res. J. Pharm. Technol..

[8] Anjum Sahair R., Sneha S., Raghu N., Gopenath T.S., Karthikeyan M., Gnanasekaran A., Chandrashekrappa G.K., Basalingappa K.M. (2018). *Solanum tuberosum* L: Botanical, Phytochemical, Pharmacological and Nutritional Significance. Int. J. Phytomed..

[9] Albishi T., John J.A., Al-Khalifa A.S., Shahidi F. (2013). Phenolic content and antioxidant activities of selected potato varieties and their processing by-products. J. Funct. Foods.

[10] Akyol H., Riciputi Y., Capanoglu E., Caboni M.F., Verardo V. (2016). Phenolic compounds in the potato and its byproducts: An overview. Int. J. Mol. Sci..

[11] Al-Weshahy A., Rao V.A. (2012). Potato Peel as a Source of Important Phytochemical Antioxidant Nutraceuticals and Their Role in Human Health—A Review. Phytochemicals as Nutraceuticals-Global Approaches to Their Role in Nutrition and Health.

[12] Xu J., Li Y., Kaur L., Singh J., Zeng F. (2023). Functional Food Based on Potato. Foods.

[13] Reddivari L., Wang T., Wu B., Li S. (2019). Potato: An Anti-Inflammatory Food. Am. J. Potato Res..

[14] Manjunath K.S., Bhandage S., Kamat S. (2015). ‘Potato Peel Dressing’: A Novel Adjunctive in the Management of Necrotizing Fasciitis. J. Maxillofac. Oral Surg..

[15] Subrahmanyam M. (2017). Treating Minor Burns with Potato Peel Dressings. J. Basic Clin. Res..

[16] Tiarasanti F., Sufiawati I., Amalia E., Sari K., Zubaedah C., Takarini V. (2024). The Effects of Potato (*Solanum tuberosum* L. vs. Granola; Solanaceae) Peel Extract Gel on Gingival Wound Healing in Wistar Rats. J. Exp. Pharmacol..

[17] Santoso A., Amalia E., Sari K., Takarini V., Sufiawati I. (2024). Histopathological Evaluation of Wound Healing and Anti-Inflammatory Effects of Granola Potato Peel Ethanol Extract in Rat Oral Mucosa. J. Exp. Pharmacol..

[18] Jimenez-Champi D., Romero-Orejon F.L., Moran-Reyes A., Muñoz A.M., Ramos-Escudero F. (2023). Bioactive compounds in potato peels, extraction methods, and their applications in the food industry: A review. CyTA-J. Food.

[19] Tierno R., Hornero-Méndez D., Gallardo-Guerrero L., López-Pardo R., de Galarreta J.I.R. (2015). Effect of boiling on the total phenolic, anthocyanin and carotenoid concentrations of potato tubers from selected cultivars and introgressed breeding lines from native potato species. J. Food Compos. Anal..

[20] Madiwale G.P., Reddivari L., Stone M., Holm D.G., Vanamala J. (2012). Combined effects of storage and processing on the bioactive compounds and pro-apoptotic properties of color-fleshed potatoes in human colon cancer cells. J. Agric. Food Chem..

[21] Thomas J., Barley A., Willis S., Thomas J., Verghese M., Boateng J. (2020). Effect of Different Solvents on the Extraction of Phytochemicals in Colored Potatoes. Food Nutr. Sci..

[22] Apel C., Lyng J.G., Papoutsis K., Harrison S.M., Brunton N.P. (2020). Screening the effect of different extraction methods (ultrasound-assisted extraction and solid–liquid extraction) on the recovery of glycoalkaloids from potato peels: Optimisation of the extraction conditions using chemometric tools. Food Bioprod. Process..

[23] Arksey H., O’Malley L. (2005). Scoping studies: Towards a methodological framework. Int. J. Soc. Res. Methodol..

[24] Levac D., Colquhoun H., O’Brien K.K. (2010). Scoping studies: Advancing the methodology. Implemment. Sci..

[25] Tricco A.C., Lillie E., Zarin W., O’Brien K.K., Colquhoun H., Levac D., Moher D., Peters M.D., Horsley T., Weeks L. (2018). PRISMA extension for scoping reviews (PRISMA-ScR): Checklist and explanation. Ann. Intern. Med..

[26] Page M.J., McKenzie J.E., Bossuyt P.M., Boutron I., Hoffmann T.C., Mulrow C.D., Shamseer L., Tetzlaff J.M., Akl E.A., Brennan S.E. (2021). The PRISMA 2020 statement: An updated guideline for reporting systematic reviews. BMJ.

[27] Guyatt G.H., Oxman A.D., Schünemann H.J., Tugwell P., Knottnerus A. (2011). GRADE guidelines: A new series of articles in the Journal of Clinical Epidemiology. J. Clin. Epidemol..

[28] Lee Y., Mohamed R.W., Yang S. (2025). Safety Profile of Solanum tuberosum-Derived Exosomes: Evidence from In Vitro Experiments and Human Skin Tests. Pharmaceuticals.

[29] Generalov E., Grigoryan I., Minaichev V., Sinitsyna O., Yakovenko L., Sinitsyn A., Generalova L. (2025). Anti-Inflammatory Effects of Solanum tuberosum L. Polysaccharide and Its Limited Gene Expression Profile. Int. J. Mol. Sci..

[30] Generalov E., Laryushkin D., Kritskaya K., Kulchenko N., Sinitsyn A., Yakovenko L., Generalova L., Belostotsky N. (2025). Immune Basis of Therapeutic Effects of Solanum tuberosum L. Polysaccharide on Chronic Peptic Ulcer Healing. Pharmaceuticals.

[31] Lee H.H., Lee S.G., Shin J.S., Lee H.Y., Yoon K., Ji Y.W., Jang D.S., Lee K.T. (2017). p-Coumaroyl Anthocyanin Mixture Isolated from Tuber Epidermis of Solanum tuberosum Attenuates Reactive Oxygen Species and Pro-inflammatory Mediators by Suppressing NF-κB and STAT1/3 Signaling in LPS-Induced RAW264.7 Macrophages. Biol. Pharm. Bull..

[32] Basilicata M.G., Pepe G., Rapa S.F., Merciai F., Ostacolo C., Manfra M., Di Sarno V., Autore G., De Vita D., Marzocco S. (2019). Anti-inflammatory and antioxidant properties of dehydrated potato-derived bioactive compounds in intestinal cells. Int. J. Mol. Sci..

[33] Rosa S.A., Adi S., Achadiyani A., Lantika U.A. (2018). The effect of Yellow Potato (*Solanum tuberosum* L.) gel on wound healing process in Mice (*Mus musculus*). Glob. Med. Health Commun..

[34] Ahmad M.F., Ahmad S.M., Kesevani R.J., Pradhan A. (2015). Anti-Ulcer Activity of Tuber Extracts of *Solanum tuberosum* (Solanaceae) in Rats. Acta Fac. Pharm. Univ. Comen..

[35] Rosas-Cruz G.P., Silva-Correa C.R., Calderón-Peña A.A., Villarreal-La Torre V.E., Aspajo-Villalaz C.L., Cruzado-Razco J.L., Del Rosario-Chávarri J., Rodríguez-Soto J.C., Pretel-Sevillano O.E., Sagástegui-Guarniz W.A. (2020). Wound Healing Activity of an Ointment from *Solanum tuberosum* L. “Tumbay Yellow Potato” on *Mus musculus* Balb/c. Pharmacogn. J..

[36] Silva-Correa C.R., Rosas-Cruz G.P., Calderón-Peña A.A., Villarreal-La Torre V.E., Aspajo-Villalaz C.L., Castañeda-Carranza J.A., Dionicio-Rosado D.Y., Gómez-Arce R.M., Rodríguez-Silva C.N., Del Rosario-Chávarri J. (2023). Effects of *Solanum tuberosum* L. ointment on second-degree burns in mice. Vet. World.

[37] Shaikh Kadar-Sha M., Shaikh K.U., Bavage N.B., Ingle P.V. (2020). A Study on Anti-Inflammatory and Anti-Ulcer Activities of Tuber Extracts of *Solanum tuberosum* (Solanaceae) By Rats. Indian Res. J. Pharm. Sci..

[38] Ahmad M.F., Ahmad S.M., Keservani R.K., Sharma A.K. (2016). Anti-inflammatory Activity of Tuber Extracts of *Solanum tuberosum* in Male Albino Rats. Nat. Acad. Sci. Lett..

[39] Silva-Beltrán N.P., Chaidez-Quiroz C., López-Cuevas O., Ruiz-Cruz S., López-Mata M.A., Del-Toro-Sánchez C.L., Marquez-Rios E., Ornelas-Paz J.D. (2017). Phenolic compounds of potato peel extracts: Their antioxidant activity and protection against human enteric viruses. J. Microbiol. Biotechnol..

[40] Helmi L., Al Khatib A., Rajha H.N., Debs E., Jammoul A., Louka N., El Darra N. (2024). Valorization of Potato Peels (*Solanum tuberosum*) Using Infrared-Assisted Extraction: A Novel Sprouting Suppressant and Antibacterial Agent. Foods.

[41] Weinberg J., Buchholz R., Parnham M., Bruinvels J. (2006). Milestones in Drug Therapy: TNF-Alpha Inhibitors.

[42] Bernatoniene J., Kopustinskiene D.M. (2018). The Role of Catechins in Cellular Responses to Oxidative Stress. Molecules.

[43] Kim J.M., Heo H.J. (2022). The roles of catechins in regulation of systemic inflammation. Food. Sci. Biotechnol..

[44] Arun K.B., Chandran J., Dhanya R., Krishna P., Jayamurthy P., Nisha P. (2015). A comparative evaluation of antioxidant and antidiabetic potential of peel from young and matured potato. Food Biosci..

[45] Al-Khayri J.M., Sahana G.R., Nagella P., Joseph B.V., Alessa F.M., Al-Mssallem M.Q. (2022). Flavonoids as Potential Anti-Inflammatory Molecules: A Review. Molecules.

[46] Choi E., Koo S. (2005). Anti-nociceptive and anti-inflammatory effects of the ethanolic extract of potato (*Solanum tuberlosum*). Food Agric. Immunol..

[47] Umadevi M., Sampath Kumar P.K., Bhowmik D., Duraivel S. (2013). Health Benefits and Cons of *Solanum tuberosum*. J. Med. Plants Stud..

[48] Polivanova O.B., Goryunova S.V., Simakov E.A., Sivolapova A.B., Zhevora S.V., Gins E.M., Mityushkin A.V. (2024). Total Phenolic, Flavonoid and Anthocyanin Content in Russian Potato. SABRAO J. Breed. Genet..

[49] Anggraini B.M.D., Herliyani D., Borneo S., Medika C., Bun P. (2018). Effect of Giving Emulgel Preparation of Kersen Leaf Extract (*Muntingia calabura* L.) and Potato Bulbs Emulgel Extract (*Solanum tuberosum* L.) with Chitosan as a Gelling Agent for Healing Burns in Rabbits. J. Borneo Cendekia.

