# Supplementary material for: Bioactive Metabolites of Solanum tuberosum L. in Inflammation Modulation and Tissue Regeneration: A Scoping Review of Wound Healing Mechanisms
Source: Biomedicines. 2026 Aug 4;14(8):1758. doi: 10.3390/biomedicines14081758 (PMC13509900; doi:10.3390/biomedicines14081758)
Supplement: Supplementary file 1 [file biomedicines-14-01758-s001.zip › biomedicines-4447521-supplementary.pdf]

Supplementary Materials

Manuscript: Bioactive Metabolites of *Solanum tuberosum* L. in Inflammation Modulation and Tissue Regeneration: A Scoping Review of Wound Healing Mechanisms

---

Table 1. PRISMA-ScR Checklist

Reporting checklist based on Tricco AC, et al. PRISMA Extension for Scoping Reviews (PRISMA-ScR). Ann Intern Med. 2018;169(7):467-473.

Item	PRISMA-ScR Item	Reported	Location in Manuscript
**Title**			
1	Identify the report as a scoping review	Yes	Title; Keywords ("scoping review")
**Abstract**			
2	Structured summary: objectives, sources, eligibility, charting methods, results, conclusions	Yes	Abstract (Background, Objective, Methods, Results, Conclusions)
**Introduction**			
3	Rationale and objectives	Yes	Introduction, paragraphs 4–6; RQ1–RQ4
**Methods**			
4	Protocol and registration	Partial	Methods 2.1; protocol not registered on PROSPERO (retrospective OSF deposition planned); Supplementary S2–S5
5	Eligibility criteria (PCC)	Yes	Methods 2.2; Table PCC
6	Information sources	Yes	Methods 2.3 (Scopus, PubMed, EBSCO, ScienceDirect; 2015–2025)
7	Search strategy	Yes	Methods 2.3; Supplementary Table S2
8	Selection process	Yes	Methods 2.4; Figure 1
9	Data charting process	Yes	Methods 2.5
10	Data items	Yes	Methods 2.5 (charting variables listed)
11	Critical appraisal	N/A	Not conducted; justified Methods 2.5 (scoping review design)

12	Synthesis methods	Yes	Methods 2.6; Table 3 evidence grading
**Results**			
13	Selection of sources	Yes	Results 3.1; Figure 1
14	Characteristics of sources	Yes	Results 3.2; Table 1 (main text)
15	Critical appraisal within sources	N/A	Not applicable
16	Results of synthesis	Yes	Results 3.3–3.6; Table 3; Discussion 4.1–4.9
**Discussion**			
17	Summary of evidence	Yes	Discussion 4.1; Conclusions
18	Limitations	Yes	Discussion 4.5 (incl. author overlap)
19	Conclusions	Yes	Section 5
**Funding**			
20	Funding	Yes	Funding section
**Conflicts of interest**			
21	Conflicts of interest	Yes	Conflicts of Interest section
**Availability of data**			
22	Availability of data, code, and other materials	Yes	Data Availability Statement

Search Strategy by Database

Search period: January 2015 – December 2025
Languages: English and Indonesian
Core concept string:

("Solanum tuberosum" OR "Solanum tuberosum L." OR potato OR "potato peel" OR "potato tuber")

AND

("wound healing" OR "wound repair" OR ulcer OR "burn wound" OR "tissue regeneration" OR "oral mucosa" OR gingiva OR "skin wound")

AND

(extract OR "bioactive metabolite*" OR phytochemical OR flavonoid OR anthocyanin OR polyphenol OR polysaccharide OR glycoalkaloid OR solanine OR chaconine OR "chlorogenic acid")

Scopus

TITLE-ABS-KEY (("Solanum tuberosum" OR potato OR "potato peel" OR "potato tuber") AND ("wound healing" OR ulcer OR "burn wound" OR "tissue regeneration") AND (extract OR "bioactive metabolite*" OR phytochemical OR flavonoid OR anthocyanin)) AND PUBYEAR > 2014 AND PUBYEAR < 2026 AND (LIMIT-TO (LANGUAGE , "English") OR LIMIT-TO (LANGUAGE , "Indonesian"))

PubMed

(("Solanum tuberosum"[Title/Abstract] OR potato[Title/Abstract] OR "potato peel"[Title/Abstract]) AND ("wound healing"[Title/Abstract] OR ulcer[Title/Abstract] OR "burn wound"[Title/Abstract] OR "tissue regeneration"[Title/Abstract]) AND (extract[Title/Abstract] OR phytochemical[Title/Abstract] OR flavonoid[Title/Abstract] OR anthocyanin[Title/Abstract] OR polysaccharide[Title/Abstract])) AND ("2015/01/01"[Date - Publication] : "2025/12/31"[Date - Publication]) AND (english[Language] OR indonesian[Language])

EBSCO (MEDLINE, CINAHL, Academic Search Complete — as available)

(TI (Solanum tuberosum OR potato OR "potato peel") OR AB (Solanum tuberosum OR potato OR "potato peel")) AND (TI ("wound healing" OR ulcer OR "burn wound" OR "tissue regeneration") OR AB ("wound healing" OR ulcer OR "burn wound" OR "tissue regeneration")) AND (TI (extract OR phytochemical OR flavonoid OR anthocyanin) OR AB (extract OR phytochemical OR flavonoid OR anthocyanin)) AND PD 20150101-20251231

ScienceDirect

("Solanum tuberosum" OR potato) AND ("wound healing" OR ulcer OR "burn wound") AND (extract OR phytochemical OR "bioactive metabolite") AND PUBYEAR > 2014 AND PUBYEAR < 2026

Iterative terms added during search refinement: inflammation, MMP-9, NF-κB, COX-2, peel extract, oral mucosa, glycoalkaloid.

Supplementary sources: Reference lists of included articles and prior potato pharmacology reviews [5,6,24]; one additional study identified by hand search.

Table 2. Evidence Grading Criteria

Evidence grades were applied within the PRISMA-ScR thematic synthesis framework [41], adapting GRADE principles of triangulation and indirectness [42]:

Grade	Criterion	Rationale
**Strong**	≥3 independent included studies with explicit molecular measurement of the same target, OR chemically defined isolate with direct pathway validation	Triangulation reduces reliance on single-laboratory findings

**Moderate**	2 independent studies, OR 1 in vivo + 1 in vitro study on the same target without third-source corroboration	Preliminary convergence; limited generalizability
**Weak**	Single study; in vitro inflammation model without wound endpoint; crude extract without molecular confirmation	High indirectness; causal attribution not established

Grades were downgraded for heterogeneous preparations, models, or metabolite classes.

End of Supplementary Materials
